# Editorial: Insights in antimicrobials, resistance and chemotherapy: 2023

**DOI:** 10.3389/fmicb.2024.1508949

**Published:** 2024-11-05

**Authors:** You-Hee Cho, Miklos Fuzi, Yuji Morita, Rustam Aminov

**Affiliations:** ^1^Department of Pharmacy, College of Pharmacy and Institute of Pharmaceutical Sciences, CHA University, Seongnam-si, Republic of Korea; ^2^Independent Researcher, Seattle, WA, United States; ^3^Department of Infection Control Science, Meiji Pharmaceutical University, Tokyo, Japan; ^4^The School of Medicine, Medical Sciences, and Nutrition, University of Aberdeen, Aberdeen, United Kingdom

**Keywords:** antimicrobial resistance, detection, epidemiology, alternatives to antimicrobials, phage therapy

Because of a significant threat posed by antimicrobial resistance (AMR), a diverse array of strategies to overcome it is currently under rigorous investigation. In the annual Research Topic “*Insights in Antimicrobials, Resistance and Chemotherapy*”, we attempt to summarize the papers that are dedicated to exploring novel developments, current challenges, recent discoveries, and future prospects within the field. This year's Research Topic includes seven contributions, with three reviews and four original research articles that cover the areas of rapid detection of AMR, epidemiology of AMR and factors contributing to the spread of AMR, and alternatives to antimicrobials.

Rapid detection of AMR, especially multidrug resistance (MDR), is important for the correct and efficient therapy. Liu et al. investigated the applicability of Oxford nanopore technology-based targeted next-generation sequencing (NanoTNGS) to determine the drug resistance profile of *Mycobacterium tuberculosis* strains. In parallel, the same strains were subjected to conventional phenotypic drug susceptibility testing and Xpert MTB/RIF assays. The sensitivity of NanoTNGS in detecting drug resistance was 93.53% for rifampicin, 89.72% for isoniazid, 85.45% for ethambutol, 74.00% for streptomycin, and 88.89% for fluoroquinolones, while specificity ranged from 83.33% to 100% for all drugs tested. Thus, NanoTNGS can rapidly and accurately determine the drug resistance profile of *M. tuberculosis* strains for timely and appropriate therapy of tuberculosis.

At the same time, the majority of prescriptions for bacterial infections are usually empirical because of the need to start the therapy as soon as possible and also for time and cost considerations associated with determining antibiotic resistance profile of a suspected bacterial pathogen. In this regard, the knowledge about the epidemiology of AMR among various pathogens in a given geographical area is important for empirical prescriptions. Almutairy summarized the current data on the epidemiology of extensively drug-resistant (XDR) and MDR bacterial strains in Saudi Arabia and some other geographical areas in the Middle East and Asia, for which the knowledge about epidemiology of AMR remains insufficient.

Factors that exert influence on differential epidemiology of AMR in various geographical locations/countries is also one of the priority areas of research. Choudhury et al. investigated the occurrence and dynamic of fluoroquinolone-resistant *Escherichia coli* (FQREC) in the USA and Iraq. In the latter country, antibiotics are frequently dispensed without prescription, and this study investigated the impact of different antibiotic use practices on the epidemiology of FQREC. In Iraq, resistance to all tested antibiotics was significantly higher, with 76.2% being FQREC, vs. 31.2% in the USA. Moreover, a significant difference in the clonal affiliation of the FQREC strains was also detected, with some minor clones of MDR strains more prominent in Iraq, while ST1193 isolates were encountered in the United States but not in Iraq.

There are also concerns that the widespread use of cationic biocides (CBs) in clinical, food chain and diverse environmental settings may contribute to the emergence of resistance to both biocides and antibiotics. Pereira et al. provided a comprehensive narrative review summarizing the responses of *Enterococcus* spp. to CBs and possible emergence of co- and cross-resistance between CBs and antibiotics. The authors identified considerable methodological and knowledge gaps and highlighted the areas of future research for better understanding the epidemiology of *Enterococcus* spp. populations subjected to the CBs exposure.

With the global rise of AMR in different pathogens, the search for alternative treatment of infections remains one of the intensively developing areas of research. This can be demonstrated by the renewed interest in phage therapy, which, however, has certain limitations such as the efficacy of phages against biofilms and the development of phage resistance. To address this problem, Ponce Benavente et al. introduced an evolutionary approach to enhance the activity of *Staphylococcus aureus* phages against biofilms. They used pre-established biofilms and real-time isothermal microcalorimetry monitoring in a serial-passage assay to obtain phages active against *S. aureus* biofilms. The phages selected demonstrated an expanded host range and higher efficacy. Thus, this approach has the potential to improve the phage therapy of biofilm-associated infections, reducing the probability of phage resistance and lessening the risk of infection relapse.

Among other antibiotic alternatives, the use of silver nanoparticles (AgNPs) have attracted considerable attention due to their broad-spectrum biocidal activities against bacteria, fungi, and viruses. The mechanisms of AgNPs activities are implemented via the generation of oxygen-reactive species, damage to DNA, rupture of bacterial cell membranes, and inhibition of protein synthesis. Rodrigues et al. summarized recent advances in this area, with the emphasis on proteomic mechanisms of antimicrobial effects of AgNPs, and how these mechanisms affect bacteria in planktonic and biofilm forms.

Many bacterial pathogens are capable of forming persister cells, with decreased metabolic activities, which significantly reduces the susceptibility to antibiotics and, therefore, efficacy of antibacterial therapy. Zhu et al. investigated the effect of the *Enterococcus faecalis* pheromone, cCF10, on the formation of biofilms and generation of persister cells. Addition of cCF10 blocked persister cell formation via metabolic mechanisms rather than inhibition of biofilm formation. The pheromone stimulated the Opp system and entered bacterial cells, inhibiting (p)ppGpp accumulation and maintaining the metabolically active state thus reducing persister cell formation.

In the annual edition of “*Insights in Antimicrobials, Resistance and Chemotherapy*”, we summarize articles that explore innovative approaches needed in antimicrobial stewardship and rapid detection, epidemiology and treatment of AMR. The 2023 edition included seven articles covering rapid detection of AMR in *M. tuberculosis*, epidemiology of AMR and factors such as prescription practices and the use of CBs that may contribute to the spread of AMR, and alternatives to antimicrobials such as phages, AgNPs, and pheromones that may help to cope with the rise of AMR. We hope that our readers would find the articles in this topic useful for their research, teaching, and public engagement.

